# Assessment of microbiological contamination in the work environments of museums, archives and libraries

**DOI:** 10.1007/s10453-015-9372-8

**Published:** 2015-03-15

**Authors:** Justyna Skóra, Beata Gutarowska, Katarzyna Pielech-Przybylska, Łukasz Stępień, Katarzyna Pietrzak, Małgorzata Piotrowska, Piotr Pietrowski

**Affiliations:** 1 Institute of Fermentation Technology and Microbiology, Lodz University of Technology, 171/173 Wólczańska St, 90-924 Lodz, Poland; 2 Institute of Plant Genetics, Polish Academy of Sciences, 34 Strzeszyńska St, 60-479 Poznan, Poland; 3 Department of Protective Equipment, Central Institute for Labour Protection – National Research Institute, 48 Wierzbowa St, 90-133 Lodz, Poland

**Keywords:** Micro-organisms at workplaces, Ergosterol, Bioaerosol, Microclimatic parameters, Museum, Archive, Library

## Abstract

Museums, archives and libraries have large working environments. The goal of this study was to determine microbial contamination in these work places and estimate the influence of microclimatic parameters and total dust content on microbial contamination. In addition, research included evaluation of ergosterol concentration and fungal bioaerosol particle size distribution. Numbers of micro-organisms in the air and on the surfaces in museums were higher (2.1 × 10^2^–7.0 × 10^3^ cfu/m^3^ and 1.4 × 10^2^–1.7 × 10^4^ cfu/100 cm^2^, respectively) than in archives and libraries (3.2 × 10^2^–7.2 × 10^2^ cfu/m^3^ and 8.4 × 10^2^–8.8 × 10^2^ cfu/100 cm^2^, respectively). The numbers of micro-organisms detected in the tested museums, archives and libraries did not exceed occupational exposure limits proposed by Polish Committee for the Highest Permissible Concentrations and Intensities of Noxious Agents at the Workplace. The concentrations of respirable and suspended dust in museum storerooms were 2–4 times higher than the WHO-recommended limits. We found a correlation between microclimatic conditions and numbers of micro-organisms in the air in the tested working environments. In addition, a correlation was also found between ergosterol concentration and the number of fungi in the air. Fungi were the dominant micro-organisms in the working environments tested. Particles within the dominant fractions of culturable fungal aerosols sampled from museum storerooms had aerodynamic diameters between 1.1 and 2.1 µm.

## Introduction

Museums, archives and libraries are institutions that are crucial for preserving cultural heritage all over the world. Given the number of such institutions, they also contribute from 0.5 to 2.6 % of total employment (based on the data of the Polish Central Statistical Office [GUS] 2012), thus constituting a significant proportion of working environments (for archivists, curators, librarians, conservators, storeroom workers and office staff). Previous microbiological analyses in museums, libraries and archives have shown high microbial air contamination, which may pose a danger to historical objects and also to staff (Gysels et al. 2004; Zielińska-Jankiewicz et al. 2008; Mesquita et al. 2009; Karbowska-Berent et al. 2011; Skóra et al. 2012).

Health threats in these institutions arise due to the inhalation of the micro-organism-contaminated air and by handling, cleaning and conserving items many of which are mould infested. Wiszniewska et al. (2009) identified allergy to fungi in 31 % of staff working at the National Museum in Warsaw (number of subjects studied n = 103). Moreover, workers’ exposure to micro-organisms may have additional consequences such as infections and mycotoxicoses. Mycotoxins produced by fungi (e.g. *Aspergillus flavus*, *A. parasiticus*, *A. versicolor*, *Penicillium chrysogenum*, *P. expansum*, *Stachybotrys chartarum)* often isolated from museums, libraries and archive facilities are known to have harmful effects (Halstensen 2008; Eduard 2009). Mycotoxins and microbial volatile organic compounds (MVOC’s) might constitute aetiological factors in sick building syndrome (SBS) (Korpi et al. 2009).

Comprehensive data describing the problem of microbial contamination in museums, libraries and archive rooms are not available. Obtaining such data is a difficult task due to the varying conditions of each institution (different collections of objects, microclimatic parameters, concentration of dust), which may impact the levels and types of micro-organisms. The goal of the present study was to determine microbial contamination in the above institutions. The study was designed to answer the following questions: Do museums, archives and libraries have similar levels and types of microbial contamination? What influence do temperature, relative humidity and concentration of dust in the air have on micro-organism numbers within these premises? Is ergosterol a good measure of fungal contamination in museums, archives and libraries? What are the risks to workers inhaling bioaerosol within these premises?

## Materials and methods

### Description of the studied rooms

Microbiological contamination was analysed in four museums, two libraries and two archives (total of 22 rooms) located in Poland. Descriptions of tested rooms are presented in Table 1. The temperature and humidity of the air in the tested rooms were determined using a PWT-401 hygrometer (Elmetron, Poland). Dust content was measured using a DustTrak DRX Aerosol Monitor (model 8533, TSI, USA). Simultaneously, measurements of size-segregated mass fraction concentrations corresponding to PM2.5 (diameter less than <2.5 μm), PM10 (<10 μm) size fractions and total dust were taken.

**Table 1:** Characteristics of the examined workplaces

Institution	Room	Cubature (m^3^)	Average temperature (°C)	Average relative humidity (%)	Total dust concentration (mg/m^3^)	Rooms description
Museum I	Restoration workshop 1	300	M: 19.5 SD: 3.8	M: 29.0 SD: 3.6	M: 0.189 SD: 0.227	Washing, drying and preliminary textile repair, no signs of moisture or moulds
	Restoration workshop 2	1200				Textile repair, no signs of moisture or moulds
	Warehouse 1	1215				Department of textile technology (“Gateway”)—collects machine (wood, steel) for processing fibres, mainly cotton and linen, no signs of moisture or moulds
	Warehouse 2	456				Historic fabric warehouse—a collection of materials on wooden and steel shelves, no signs of moisture or moulds
Museum II	Warehouse 1	281	M: 19.0 SD: 1.0	M: 41.0 SD: 3.6	M: 0.131 SD: 0.030	Collection of paintings on gantries, weapons (swords, rifles, pistols), explicit signs of moisture and moulds
	Warehouse 2	220				Collection of flags, banners gathered in wooden dressers, explicit signs of moisture and moulds
	Warehouse 3	52				Collection of flags, banners gathered in wooden dressers, explicit signs of moisture and moulds
Museum III	Warehouse 1	310	M: 13.3 SD: 3.1	M: 39.7 SD: 4.7	M: 0.373 SD: 0.105	Furniture warehouse, department of folk art—collection of wooden furniture, sculptures, some signs of moisture on objects and walls
	Warehouse 2	1362				Section of farm and rural industry—collection of materials such as wood, wicker, iron, derived mostly from the second half of nineteenth and twentieth centuries, no signs of moisture or moulds
	Warehouse 3	60				Magazine of African cultures—two part room, collection of clothing, masks, weapons, sculptures no signs of moisture or moulds
Museum IV	Warehouse 1	222	M: 22.0 SD: 0.8	M: 35.5 SD: 11.1	nt	Cabinet of polish prints and drawing, no signs of moisture or moulds
	Warehouse 2	725				Art warehouse—collection of oil paintings on canvas and board, no signs of moisture or moulds
	Warehouse 3	171				Mediaeval art restoration workshop—work only with wood, no signs of moisture or moulds
	Exhibition hall	3055				Ancient art gallery—collection of sculptures from this period, no signs of moisture or moulds
Archive I	Warehouse 1	222	M: 22.0 SD: 1.0	M: 36.2 SD: 3.2	nt	The oldest room gathers records of Łódź since 1884 on metal shelves. The files are packed in protective cartons, no signs of moisture or moulds
	Warehouse 2	236				Books and map of Łódź and its region, archival photographs, windows in the room are covered in order to reduce exposure of sets, no signs of moisture or moulds
Archive II	Warehouse 1	405	M: 19.3 SD: 1.5	M: 31.5 SD: 7.4	M: 0.073 SD: 0.011	Files, maps and books from nineteenth century factories, court records from nineteenth to twentieth centuries no signs of moisture or moulds
	Warehouse 2	176				Files maps and books from nineteenth century factories, court records from nineteenth to twentieth centuries no signs of moisture or moulds
Library I	Warehouse 1	222	M: 23 SD: 0.8	M: 51.4 SD: 7.4	M: 0.158 SD: 0.103	Metal shelves with books, room sealed, new plastic window, no signs of moisture or moulds
	Warehouse 2	69				Basement, wooden bookshelves, lack of ventilation, signs of water damage on the walls, flaky paint, destroyed by moulds book on the floor, no signs of moisture or moulds
Library II	Warehouse 1	123	M: 18.5	M: 27.3	M: 0.035	Books from nineteenth to twentieth centuries, no signs of moisture or moulds
	Warehouse 2	138	SD: 0.7	SD: 1.0	SD: 0.006	Books and periodicals from nineteenth to twentieth centuries, no signs of moisture or moulds
Office rooms in the tested institutions		52–78	M: 21.6 SD: 2.3	M: 37.0 SD: 11	M: 0.108 SD: 0.049	Office equipment, cabinets and shelves for documents, no signs of moisture or moulds

*M* arithmetic mean; *SD* standard deviation; *nt* not tested

### Determination of microbiological contamination of air and surfaces

Air Sampler MAS-100 Eco (Merck, Germany) was used for air sampling. Air samples of 50 and 100 L were taken on DG18 agar medium (Dichloran Glicerol Selective Medium, Merck, Germany) and MEA medium (Malt Extract Agar, Merck, Germany) with chloramphenicol (0.1 %) for determining total fungal number (including xerophilic and hydrophilic fungi) and on a TSA medium (Tryptic Soy Agar, Merck, Germany) with nystatin (0.2 %) for determining total bacterial number. Air samples were collected in two sequential repetitions on each medium, in three locations in each room using a single sampler. Samples were collected in winter 2011 (between November and February), during one working day, when staff were performing routine activities in the tested museums, archives and libraries.

Office rooms were analysed as internal backgrounds for each site (three repetitions on each medium), and the atmospheric air outside each building site was analysed as the external background (three repetitions on each medium). Bioaerosol particle sizes were determined from samples collected in rooms with the highest level of fungal contamination (storerooms in Museum II), since they might pose the greatest health risk to employees. Samples for determining particle size distribution of fungal bioaerosol were collected from selected museum storerooms, an office room (internal background) and atmospheric air (external background), using a six-stage Andersen sampler (model WES-710, Westech Instruments, UK). Samples were collected over 5 min (141.5 l of air) on MEA medium with chloramphenicol (0.1 %) in two repetitions on each medium in three places in room.

Samples from surfaces were collected using Envirocheck^®^ plates (Merck, Germany) on TSA medium with neutralizers (for bacteria) and on Sabouraud medium (for fungi). For highly contaminated surfaces, the traditional swab method was applied, using saline solution (0.85 % NaCl), swabs, metal frames of 25 cm^2^ surface area and the media described above. Samples were collected from 3 to 5 surfaces (furniture, walls, books and stored objects) on each medium, in every room (total number of samples in a given institution: 10–15). The samples were incubated at 30 ± 1 °C for 48 h (bacteria) or at 27 ± 1 °C for 5 days (fungi). After incubation, the colonies were counted, and the results were expressed in cfu/m^3^ (air) or cfu/100 cm^2^ (surfaces). The final results are presented as the arithmetic mean of all repetitions.

### Identification of bacteria and yeasts

All bacteria and yeasts isolated from samples were transferred onto individual culture plates. Following this, they were macroscopically and microscopically characterized using Gram-staining, catalase test and oxidase test (Microbiologie Bactident Oxydase, Merck, Germany). Next, isolates with the same morphology and biochemical features were grouped into strains and identified using API tests (BioMérieux, France): API 50 CH, API STAPH and API 20 NE (for bacteria) and API C AUX (for yeasts). Identified bacteria were genetically confirmed using the 16S rRNA gene nucleotide sequence (Jensen et al. 1993).

Isolated filamentous fungi were cultured on CYA (Czapek Yeast Extract Agar, Difco, USA) and YES medium (yeast extract with supplements) and visually identified, macroscopically and microscopically, using taxonomic keys (Bensch et al. 2012; Frisvad and Samson 2004; Pitt and Hocking 2009; Klich 2002). Identity of moulds and yeasts was confirmed using ITS1/2 sequence of the rDNA region (White et al. 1990). Genomic DNAs were extracted using a method described previously (Stępień et al. 2011). The resulting nucleotide sequences of the studied micro-organisms were analysed and compared to the sequences published in the National Center for Biotechnology Information (NCBI) database, using the BLASTN 2.2.27+ program (Zhang et al. 2000).

### Ergosterol determination

Air samples for ergosterol determination were collected using an AirPort MD8 sampler (Sartorius, Germany). In total, 1000-L samples (six repetitions) were collected on sterile gelatine filters (pore diameter 0.3 µm, Sartorius, Germany). Ergosterol was quantified based on Miller and Young’s (1997) method using a modified analytical procedure. Chromatographic analysis (gas chromatography with flame ionization detection—GC-FID) was carried out using a GC apparatus (Agilent 6890N HP, USA). Quantitative analysis was performed using an external standard (external calibration) method, with software from Agilent ChemStation (USA).

### Statistical analyses

Statistical analyses were performed using STATISTICA 6.0 software (Statsoft, USA). All results were evaluated using one-way analysis of variance (ANOVA) at the 0.05 significance level. When statistical differences were detected (*p* < 0.05), means were compared by the post hoc Fisher’s test at 0.05 significance level.

Linear regression analysis was used to determine the effect of air humidity and temperature and total dust on microbiological contamination of the air and surfaces in tested buildings. Linear regression analysis was also used to determine the effect of air and surface microbiological contamination on ergosterol concentration in the air. The significance tests were performed at the 0.05 significance level using Guilford’s correlation scale (Stanisz 2006).

## Results

**Fig. 1:**
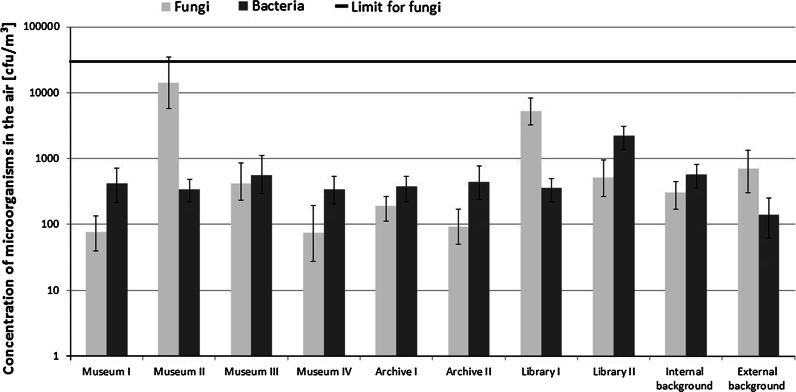
Mean concentration (± 1 SD) of micro-organisms in museums, archives and libraries, including internal (office) and external (outdoors) background. Number if samples (N = 12–24). Limit for fungi—occupational exposure limits proposed by polish committee for the highest permissible concentrations and intensities of Noxious Agents at the workplace

**Fig. 2:**
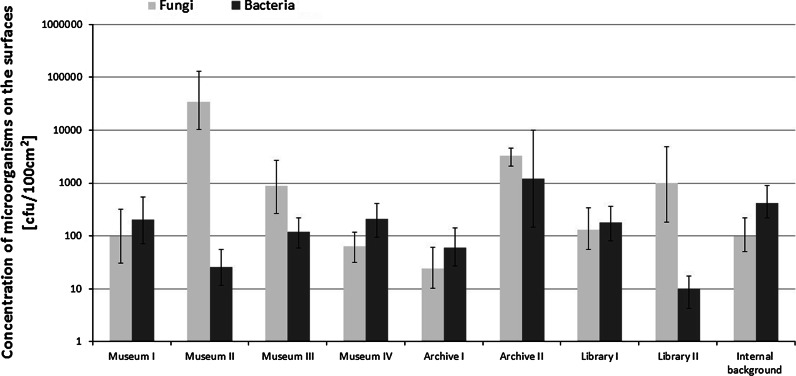
Mean concentration (± 1 SD) of micro-organisms on the surface in museums, archives and libraries including internal (office) background. Number of samples (N = 12–24)

The number of micro-organisms in the air in museums averaged from 2.1 × 10^2^ to 7.0 × 10^3^ cfu/m^3^ and on the surfaces from 1.4 × 10^2^ to 1.7 × 10^4^ cfu/100 cm^2^ (Figs. 1, 2). Museum IV had the lowest level of air and surface microbiological contamination (high standards of hygiene, monitoring system for microclimatic parameters, museum of national rank). The highest concentration of bacteria was found in the air of Museum III, whose collections consist of folk culture objects (mainly made of wood and fabric). The highest statistically significant (*p* < 0.05) fungal contamination was found in Museum II, a war memorial museum (collections of paintings, firearms, weapons and flags). The fungal concentration in the rooms of that museum was 20 times greater than in the atmospheric air (*p* < 0.05).

The level of microbial contamination in the air of archives and libraries ranged from 4.9 × 10^2^ to 5.6 × 10^3^ cfu/m^3^, while the number of surface micro-organisms was between 8.4 × 10^1^ and 3.9 × 10^3^ cfu/100 cm^2^. Amongst institutions of this type, the highest microbiological air contamination was found in Library I (rooms with signs of moisture and moulds on walls and books). The air of this library had higher concentrations of airborne fungi compared to other similar institutions (5.3 × 10^3^ cfu/m^3^, *p* < 0.05).

The number of micro-organisms on the surfaces was low, and the collected materials in most institutions did not have active microbial growth; except Museum II, Archive II had high levels of contamination on objects. However, there were no statistically significant differences in the levels of fungi (except Museum II) and bacteria (except Archive II) on the surfaces in the tested buildings (*p* > 0.05).

**Fig. 3:**
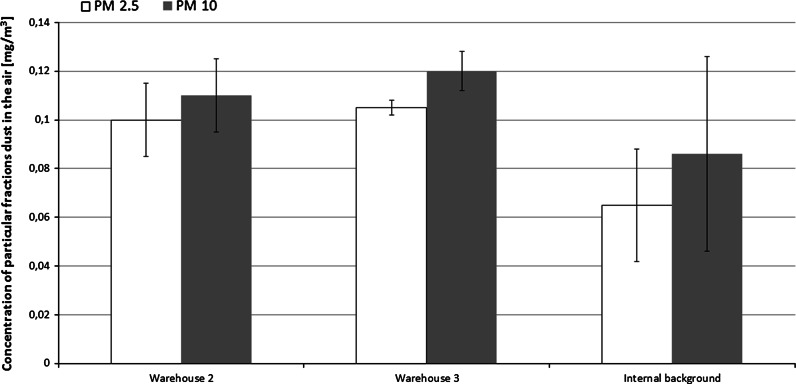
Mean concentration (± 1 SD) of ergosterol concentration in the air in tested institutions including internal (office) background. Number of samples (N = 3–6)

The concentrations of ergosterol ranged from 0.41 to 0.69 ng/m^3^ (Fig. 3). The highest level of ergosterol was found in Museum III and Library II, which may indicate problems with fungal infestation. The lowest concentration of ergosterol (0.41–0.46 ng/m^3^) was found in archive rooms.

Regression analysis revealed high positive correlations between ergosterol concentrations and numbers of fungi in the air in museums (*r* = 0.787–0.829, *p* < 0.05) and archives (*r* = 0.776–0.939, *p* < 0.05). Results of regression analysis undertaken for libraries indicate a moderate influence of fungal contamination on ergosterol concentration in Library I (*r* = 0.482, *p* < 0.05); however, the significance coefficient calculated for Library II (*r* = 0.311, *p* > 0.05) shows no correlation between the examined values.

The concentration of ergosterol in the air and the number of fungi on the surfaces in two workplaces had moderate correlation: Museum I (*r* = 0.490, *p* < 0.05) and Archive I (*r* = 0.461, *p* < 0.05). All other institutions examined showed no correlation between these two parameters (significance levels *p* > 0.05). The influence of microclimate parameters on the number of micro-organisms in the air was tested. The correlation between the temperature and number of micro-organisms in air samples was high (*r* = 0.610–0.863, *p* < 0.05) in 7/8 institutions. Regression coefficients describing the effect of air humidity on microbial concentration showed high correlations (*r* = 0.690–0.971, *p* < 0.05) in 6/8 institutions.

Significant correlations (*p* < 0.05) were also observed between microclimatic parameters and the number of surface micro-organisms in 6/8 institutions; correlation coefficient (*r*) equalled from 0.412 to 0.890 and from 0.428 to 0.906 for temperature and humidity, respectively. No significant correlations (*p* > 0.05) were found in two institutions (Library II and Museum IV).

The impact of total dust concentrations on air microbial contamination levels was determined. In museums and libraries, linear regression results showed high correlation between these parameters. This was confirmed by high correlation coefficients, which ranged from 0.794 to 0.908 (*p* < 0.05) for all workplaces except Archive 1. Correlation in Archive 1 was not significant (*r* = 0.351, *p* > 0.05).

**Fig. 4:**
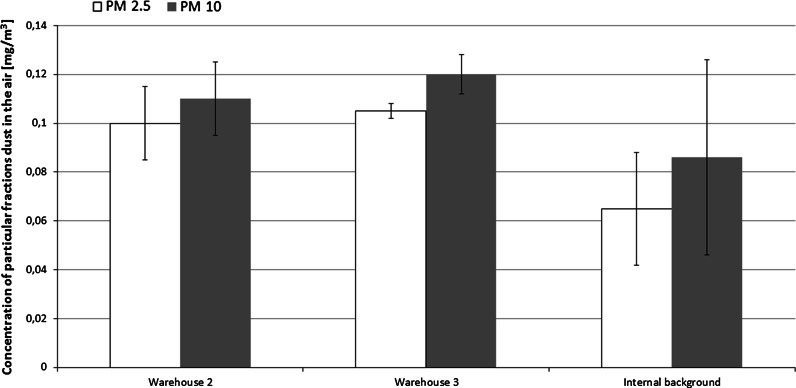
Mean concentration (± 1 SD) of particular dust fraction in the air in tested institutions including internal (office) background. Number if samples (*N* = 12–24). PM2.5—respirable dust with a diameter less than 2.5 μm; PM10—particulate matter with a diameter less than 10 μm (mouth and nose)

Moreover, the concentration of respirable dust (particle diameter <2.5 µm) in museum storerooms ranged between 0.100 and 0.105 mg/m^3^, which was higher than office rooms (internal background samples) (Fig. 4).

**Fig. 5:**
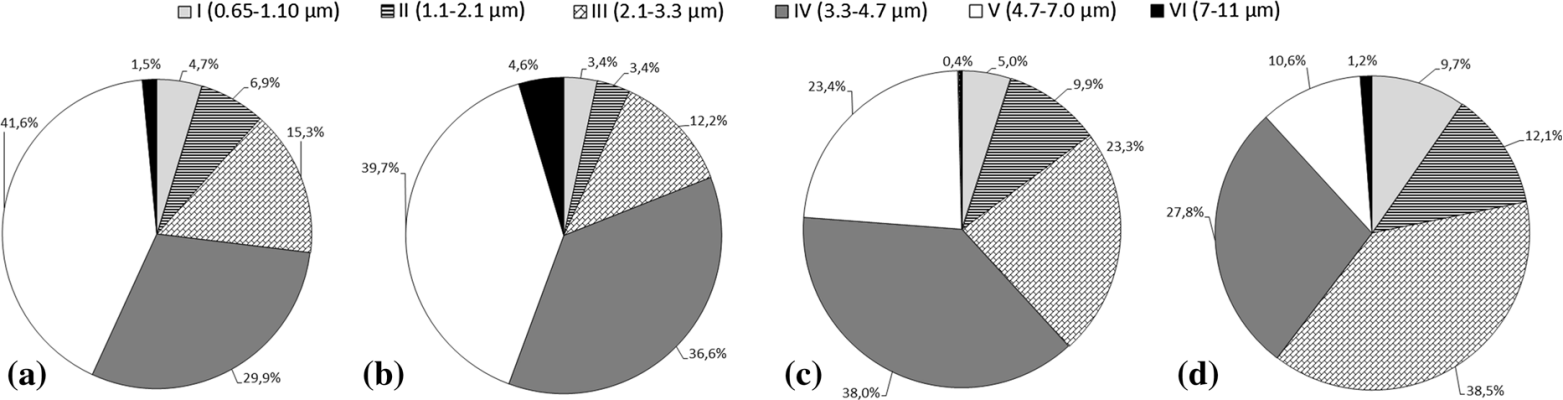
Percentage contribution of each size fraction (aerodynamic diameter I–VI) of fungal bioaerosol. **a** Warehouse 2 in Museum II; **b** warehouse 3 in Museum II; **c** internal background (office); **d** external background (outdoors). Number of samples (N = 6)

The dominant fraction of culturable fungal aerosol in museum storerooms had aerodynamic diameters between 1.1 and 2.1 µm (fraction V), and it accounted for 40–42 % of the fungal bioaerosols, depending on the room. One of the storerooms (Storeroom 3) had a large contribution from fraction IV (36 %), consisting of particles with aerodynamic diameters of 2.1–3.3 µm. This was also the dominant fraction culturable fungal aerosol in samples from office rooms (internal background), while the greatest percentage contribution from atmospheric air (external background) came from particles measuring 3.3–4.7 µm (fraction III) (Fig. 5).

A total of 16 bacterial strains were isolated from museum premises, 13 from archives and 13 from libraries (Table 2). Bacteria from the *Micrococcus,* *Staphylococcus* and *Bacillus* genera dominated each institution type, while other species of bacteria were specific to particular buildings. Qualitative composition of fungi varied in tested environments: 32 species of fungi (28 moulds and 4 yeasts) were isolated in museums, 24 species (21 moulds and 3 yeasts) in archives and 34 (32 moulds and 2 yeasts) in libraries (Table 2). The most common fungi were as follows: *Aspergillus puulaaensis, Cladosporium cladosporioides, Penicillium crustosum, Rhizopus oryzae.* However, there were distinct differences in air and surface fungal composition between institutions. It is worth emphasizing that 45 amongst the 89 isolated micro-organisms are potential pathogens according to classifications of the Directive UE 2000/54/WE, Regulation of the Minister of Health in Poland dated 22 April 2005, the European Confederation of Medical Mycology (BSL) and the Institute of Rural Health in Lublin (IMW). These belong to the genera: *Bacillius, Pseudomonas, Alternaria, Aureobasidium, Aspergillus, Chaetomium, Chrysonilia, Cryptococcus, Cladosporium, Paecilomyces, Mucor, Penicillium, Talaromyces* and *Trichoderma* (Table 2).

**Table 2:** Isolated species and frequency of their isolation in the air and on the surfaces in the examined institutions

Micro-organism	Frequency of isolation of all samples in each institution [%] Air/surfaces							
	Museum I	Museum II	Museum III	Museum IV	Archive I	Archive II	Library I	Library II
**Bacteria**								
*Aneurinibacillus aneurinilyticus*	0/0	0/0	0/0	0/0	0/0	0/0	0/0	0/33.3
*Bacillus circulans*	0/0	0/0	0/0	0/0	0/0	0/0	22.2/0	0/0
*Bacillus firmus*	75.0/0	0/0	0/0	0/0	0/0	0/0	0/0	0/0
*Bacillus licheniformis*	0/0	0/0	0/0	0/0	0/0	21.4/0	0/0	8.3/33.3
*Bacillus megaterium*	0/0	0/0	0/58.3	0/0	0/41.7	0/0	0/0	0/0
*Bacillus mycoides*	0/0	0/0	0/0	0/0	0/0	0/0	0/80	0/0
***Bacillus pumilus***	0/55.0	0/0	0/75.0	0/62.5	0/0	21.4/26.3	0/45	8.3/0
*Bacillus* sp.	0/66.7	0/0	0/0	0/0	0/0	0/0	0/0	0/0
***Bacillus subtilis***	0/44.4	0/0	0/66.7	0/0	0/0	21.4/10.5	0/0	33.3/33.3
*Brevibacillus laterosporus*	0/50.0	0/0	0/0	0/0	0/0	0/0	0/0	0/0
*Kocuria kristinae*	0/0	0/0	0/0	0/0	0/0	0/0	22.2/0	0/0
*Kocuria varians*/*rosea*	75.0/0	0/0	0/0	0/0	0/0	0/0	0/0	0/0
*Lactobacillus delbrueckii*	0/50.0	0/50.0	0/0	0/0	0/0	0/0	0/0	0/0
*Leuconostoc mesenteroides*	0/0	0/0	0/0	0/0	61.1/0	0/0	0/0	0/0
*Micrococcus flavus*	0/0	0/0	0/0	0/0	0/0	92.9/0	0/0	0/0
*Micrococcus* sp.	95.9/83.3	100.0/0	86.1/16.7	95.9/56.3	100/41.7	0/0	66.7/0	100/0
*Micromonospora* sp.	0/0	0/0	0/0	0/0	0/0	0/26.3	0/0	0/0
*Nocardia* sp.	0/0	0/0	0/0	0/0	0/0	21.4/0	0/0	41.7/0
*Paenibacillus polymyxa*	0/0	0/0	0/91.7	0/0	0/0	0/0	0/0	0/0
***Pseudomonas alcaligenes***	0/0	75.0/0	0/0	0/0	0/0	0/0	22.2/0	0/0
***Pseudomonas stutzeri***	0/0	0/0	0/0	0/0	0/0	0/0	27.8/0	0/0
***Pseudomonas oryzyhabitans***	0/0	0/0	0/0	0/0	38.9/0	0/0	0/0	0/0
*Sphingomonas paucimobilis*	0/0	0/0	0/0	0/50.0	0/0	0/0	0/0	0/0
*Staphylococcus haemolyticus*	0/0	0/0	100.0/0	0/0	0/0	0/0	0/0	0/0
*Staphylococcus cohnii*	0/0	0/0	0/0	0/0	94.4/0	0/0	0/0	0/0
*Staphylococcus hominis*	91.7/0	0/0	0/0	0/0	0/0	0/0	0/0	0/0
*Staphylococcus lentus*	0/0	100.0/0	0/0	0/0	0/0	0/26.3	100/0	66.7/0
*Staphylococcus xylosus*	75.0/0	0/0	0/0	0/0	0/0	0/21	0/0	33.3/0
**Fungi**								
*Acremonium furcatum*	0/0	0/0	0/0	0/0	5.6/0	0/0	0/0	0/0
*Acremonium verticillium*	0/0	0/0	0/0	0/0	0/0	0/0	11.1/0	0/0
***Alternaria alternata***	0/14.3	0/0	0/0	0/43.8	0/0	7.1/0	0/35	0/0
*Alternaria consortiale*	0/14.3	0/0	0/0	0/0	0/0	0/0	5.6/0	16.7/0
*Alternaria* sp.	0/0	0/0	0/0	0/0	0/0	14.3/0	0/0	0/0
*Alternaria tennuissima*	0/0	0/0	0/0	0/0	0/0	0/0	33.3/15	0/0
***Aspergillus clavatus***	0/0	0/0	0/0	0/0	0/0	0/0	5.6/0	0/0
***Aspergillus flavus***	0/0	0/0	0/0	0/12.5	0/0	0/0	0/0	0/12.5
***Aspergillus fumigatus***	0/0	0/0	0/0	0/0	0/0	7.1/0	0/0	0/0
***Aspergillus niger***	20.8/28.6	0/12.5	0/0	4.2/0	0/0	7.1/0	16.7/15	8.3/0
***Aspergillus ochraceus***	0/0	0/0	0/0	8.3/0	0/0	0/0	0/15	0/0
***Aspergillus parasiticus***	0/0	0/0	0/27.3	0/0	0/0	0/0	0/0	0/0
***Aspergillus puulaaensis***	25.0/0	0/50.0	61.1/0	4.2/0	0/0	14.3/11.1	0/0	58.3/0
***Aspergillus terreus***	0/0	0/0	0/0	12.5/0	0/0	0/0	0/0	0/0
*Aspergillus wentii*	0/0	0/0	0/0	8.3/0	0/0	0/0	0/0	0/0
***Aureobasidium pullulans***	0/0	0/0	0/0	4.2/0	0/0	0/0	0/0	0/0
*Beauveria* sp.	0/0	0/0	0/0	0/0	11.1/0	0/0	0/0	0/0
*Botrytis cinerea*	0/0	0/0	0/0	0/0	5.6/0	0/0	16.7/0	0/0
*Botrytis* sp.	4.2/0	0/0	0/0	0/0	0/0	7.1/0	0/0	8.3/0
***Chaetomium globosum***	0/0	0/0	0/0	0/0	0/0	0/0	0/10	0/0
***Chrysonilia sitophila***	0/0	0/0	0/0	0/0	38.9/0	21.4/0	0/0	0/0
***Cladosporium cladosporioides***	0/0	0/12.5	38.9/0	0/12.5	16.7/12.5	14.3/33.3	72.2/35	58.3/0
***Cladosporium herbarum***	0/0	0/0	50.0/0	16.7/18.8	72.2/0	7.1/0	0/30	58.3/0
*Cladosporium* sp.	4.2/0	0/0	0/0	0/0	0/0	0/0	0/0	0/0
*Epicoccum nigrum*	0/0	0/0	0/0	0/0	0/0	0/0	16.7/0	0/0
*Eurotium amstelodami*	0/0	0/0	0/0	0/0	0/0	0/0	0/25	0/0
*Gliocladium* sp.	0/0	0/0	0/0	0/0	0/0	0/0	5.6/0	0/0
***Mucor circinelloides***	0/0	0/0	0/0	0/6.3	0/0	0/0	0/10	0/12.5
***Mucor globosus***	0/0	0/0	0/0	0/0	0/0	0/0	0/0	8.3/0
***Mucor hiemalis***	8.3/0	0/0	0/0	0/0	0/0	0/0	0/0	0/0
***Mucor plumbeus***	0/0	0/0	0/18.9	0/0	0/0	0/0	0/0	0/0
***Mucor racemosus***	0/14.3	0/0	0/18.9	0/0	0/0	0/0	0/0	0/0
***Mucor*** ** sp.**	0/0	0/0	0/0	0/0	0/0	0/0	0/0	8.3/0
***Paecilomyces variotii***	0/0	0/0	0/0	0/31.3	0/0	0/44.4	0/0	0/0
***Penicillium griseofulvum***	0/0	0/0	0/0	0/0	0/0	0/0	11.1/0	0/0
***Penicillium chrysogenum***	0/7.1	0/25.0	0/0	8.3/18.8	0/0	0/0	27.7/20	0/0
***Penicillium commune***	0/0	0/0	0/0	0/0	0/0	0/0	16.7/0	0/0
***Penicillium corylophilum***	0/0	0/0	0/27.3	25.0/18.8	0/0	0/0	0/0	8.3/0
***Penicillium crustosum***	45.8/57.1	0/12.5	5.6/36.4	0/0	0/0	28.6/11.1	33.3/0	33.3/50
***Penicillium freii***	0/0	0/0	0/0	0/0	61.1/0	21.4/33.3	0/0	50/25
***Penicillium gladioli***	0/0	0/0	0/0	20.8/0	0/0	0/0	0/0	0/0
***Penicillium hirsutum***	0/0	0/0	0/0	0/0	5.6/0	0/0	0/0	0/0
***Penicillium janthinellum***	0/0	0/0	0/0	4.2/62.5	0/0	14.3/11.1	0/0	0/0
***Penicillium olsonii***	0/0	0/0	0/0	0/0	0/25.3	0/0	0/0	0/0
***Penicillium oxalicum***	0/0	0/0	0/0	0/0	5.6/0	0/0	0/0	0/0
***Penicillium radicola***	45.8/0	0/0	0/0	0/0	0/0	0/0	0/0	0/0
***Penicillium sclerotigenum***	0/0	0/0	0/0	8.3/0	0/0	0/0	0/0	0/0
***Penicillium viridicatum***	0/0	0/0	0/0	0/0	16.7/0	0/0	0/0	0/0
***Penicillium waksmanii***	0/0	0/0	0/0	0/0	0/0	0/0	55.6/0	0/0
***Rhizopus oryzae***	37.5/14.3	100/0	27.8/0	0/0	38.9/56.3	14.3/0	0/0	0/12.5
***Talaromyces wartmannii***	0/0	0/0	0/0	0/0	0/0	0/0	0/10	0/0
*Trichoderma koningii*	0/0	0/0	0/0	0/0	0/0	0/0	22.2/20	0/0
***Trichoderma viride***	0/0	0/0	5.6/0	0/0	0/0	0/0	0/25	0/0
*Ulocladium* sp.	0/0	0/0	0/0	0/0	0/0	0/0	5.6/0	0/0
**Yeast**								
*Candida* sp.	0/0	0/0	0/0	0/31.3	0/0	0/0	0/0	0/0
*Candida sphaerica*	0/35.7	0/0	0/0	0/0	0/0	0/0	0/0	0/12.5
*Cryptococcus humicola*	0/0	0/0	0/0	0/0	0/18.8	0/0	0/0	0/0
***Cryptococcus laurentii***	0/0	0/0	0/0	0/0	0/0	0/0	22.2/0	0/0
*Cryptococcus uniguttulatus*	0/0	0/0	0/0	0/0	0/6.3	0/0	0/0	0/0
*Rhodotorula* sp.	0/0	0/0	0/9.1	0/0	0/0	0/11.1	0/0	0/0
*Rhodotorula minuta*	0/0	0/0	0/0	0/6.3	0/0	0/0	0/0	0/0

Species that can constitute occupational threat according to the classification of the Directive UE 2000/54/WE, Regulation of the Minister of Health in Poland dated 22 April 2005, the European Confederation of Medical Mycology (BSL) and the Institute of Rural Health in Lublin (IMW) are bolded

## Discussion

The buildings studied differed from each other in air contamination levels, which may be due to specific features and the condition of housed collections. Tested institutions had similar levels of surface microbial contamination (furniture, walls, stored objects). The levels of microbiological contamination in all institutions studied were lower than the threshold values of occupational exposure specified by the Polish Committee for the Highest Permissible Concentrations and Intensities of Noxious Agents at the Workplace, which is 5.0 × 10^4^ cfu/m^3^ for the total fungal count (Skowroń and Górny 2012).

We found lower or similar microbial contamination in archives compared to previous studies (Borrego et al. 2010; Karbowska-Berent et al. 2011) and higher contamination in libraries and museums (Camuffo et al. 2001; Niesler et al. 2010; Karbowska-Berent et al. 2011).

Low ergosterol levels (0.41–0.69 ng/m^3^) in the air of studied rooms confirmed observations made by Gutarowska et al. (2014) for rooms with low fungal contamination. An ergosterol concentration above 1 ng per m^3^ can be considered as an indicator of excessive mould contamination of indoor air (10^3^ cfu/m^3^) (Gutarowska et al., 2014). Our study found a significant correlation between the number of fungi in the air of museums and archives and ergosterol concentration. Ergosterol has been recommended previously as a good chemical indicator for evaluating mould concentrations in the air (Miller and Young 1997; Pasanen et al. 1999; Robine et al. 2005). No correlations were found between the number of fungi on the surfaces and ergosterol concentrations.

Steady microclimatic parameters, temperature of 20 ± 2 °C and relative air humidity of 50 ± 3 %, are recommended for collection storage in the studied institution types (ISO 11799:^2003^; Schäfer 2014). The temperature in 2/8 and the humidity in 7/8 institutions deviated from those specified in the standard. This was probably due to the lack of ventilation and air conditioning systems. There was an effect of humidity and temperature on microbial concentration in the air in majority of the facilities tested.

The highest concentration (0.38–0.39 mg/m^3^) of respirable dust, with particle sizes below 4 µm, was recorded in the rooms of Museum III. This concentration was 2–4 times higher than the 24-h exposure limits specified in the World Health Organization recommendations (WHO 2005). Fractions with aerodynamic diameters of 1.1–2.1 µm had the highest percentage contribution in fungal aerosols sampled from museum storerooms (Museum II). Based on spore sizes of the most frequently isolated fungal species, *Aspergillus* (2.1–3.6 µm), *Penicillium* (2.8–5.0 µm) and *Cladosporium* (2.5–7.5 µm), we conclude that fungi occurred as single cells, as spores or small fragments of mycelium.

Based on the dust level and particle size distribution data, the aerosol within the studied workplaces is able to penetrate the human respiratory system. This can happen via nasal and oral cavities, primary, secondary and terminal bronchi and the pulmonary bronchioles, with the solid particles present in dust acting as carriers. This may cause irritation of the mucous membranes of the nose and eyes, and an inflammatory response or allergic reactions (Kulkarni et al. 2011), in exposed employees.

Commonly detected micro-organisms in the tested institutions included: *Micrococcus* sp., *Staphylococcus* sp., *Bacillus* sp.*, Aspergillus puulaaensis, Cladosporium cladosporioides, Penicillium crustosum, Rhizopus oryzae.* Previous studies by Karbowska-Berent et al. (2011), Niesler et al. (2010) have shown, inter alia, that these micro-organisms colonize museums, libraries and archival facilities.

Finally, most of the identified micro-organisms constitute an occupational threat according to the literature (Report of a Working Group on Hazardous Fungi of the European Confederation of Medical Mycology 1996; Directive UE 2000/54/WE, Regulation of the Minister of Health in Poland dated 22 April 2005, Dutkiewicz et al. 2007).

Factors such as dust concentration, diameter of fungal bioaerosol particles and the presence of potentially pathogenic micro-organisms should be taken into account in the microbiological assessment of working environments in museums, archives and libraries. Those factors may affect the health of workers; however, it requires further study.

## Conclusions

The levels and types of micro-organisms within museums, archives and libraries were institution dependent. Micro-organism numbers within workspaces did not exceed recommended limits for occupational exposure. The concentrations of respirable and suspended dust in museum storerooms were 2–4 times higher than the WHO-recommended limits. Fungi were the dominant group of micro-organisms within tested working environments. The dominant fungal aerosol fractions had aerodynamic diameters that ranged between 1.1 and 2.1 µm in museum storerooms. We found a correlation between relative humidity and temperature and microbial contamination in the air. The concentration of ergosterol in the air in museum, archive and library workplaces strongly correlates with fungal concentrations in the air.
